# Medicare Coverage and Patient Out-of-Pocket Costs for Cardiovascular-Kidney-Metabolic Medications

**DOI:** 10.1001/jamanetworkopen.2024.12437

**Published:** 2024-05-21

**Authors:** Grant M. Young, Kannu Bansal, Ralph J. Riello, Katherine A. A. Clark, Sanket S. Dhruva, Kamil F. Faridi, Nihar R. Desai

**Affiliations:** 1 Section of Cardiovascular Medicine, Department of Internal Medicine, Yale School of Medicine, New Haven, Connecticut; 2 Department of Internal Medicine, Saint Vincent Hospital, Worcester, Massachusetts; 3 Section of Cardiology, Department of Medicine, University of California San Francisco School of Medicine, San Francisco

## Abstract

This cross-sectional study evaluates the association between Medicare coverage and patient out-of-pocket costs for cardiovascular-kidney-metabolic medications.

## Introduction

The American Heart Association recently released an advisory statement outlining the importance of addressing cardiovascular-kidney-metabolic (CKM) health.^1^ One in 2 Medicare beneficiaries have at least 1 CKM condition and 1 in 4 have overlapping CKM conditions.^2^ Understanding health care delivery challenges such as lack of insurance coverage and high out-of-pocket (OOP) costs is critical to improving management and disparities in CKM conditions. This study aimed to provide a contemporary analysis of coverage, access restrictions, and OOP costs for CKM therapies under Medicare Part D plans in 2023.

## Methods

Medicare Prescription Drug Plan Formulary and Pricing files from quarter 3 (July-September) of 2023 were used to study medication classes, including glucagon-like peptide-1 receptor agonists (GLP1RA), tirzepatide (combined glucose-dependent insulinotropic polypeptide [GIP]/GLP1RA), sodium-glucose-cotransporter-2 inhibitors (SGLT2i), and nonsteroidal mineralocorticoid receptor antagonist (nsMRA). Plan coverage and restrictions (prior authorization, step therapy, and tier level ≥3 cost sharing) were analyzed. Annual OOP costs were estimated for each plan covering a particular therapy using the 2023 Medicare Part D benefit (eMethods in Supplement 1).^3^ The analyses were conducted using RStudio version 4.3.1 (R Project for Statistical Computing). This study was exempt from institutional review board review and the need for informed consent since it used publicly available data and did not include human participants. This study followed the Strengthening the Reporting of Observational Studies in Epidemiology (STROBE) reporting guideline reporting guidelines.

## Results

There were a total of 4754 plans. GLP1RA had widespread coverage (4073 plans [85.7%] to 4675 plans [98.3%]), apart from short-acting exenatide (2694 plans [56.7%]). Only 2392 plans (50.3%) covered tirzepatide. Nearly all GLP1RA and GIP/GLP1RA medications were restricted (97.9%-99.8%) through tier level 3 or higher (97.3%-98.7%), prior authorization (14.3%-26.9%), or step therapy (6.4%-10.2%). Among SGLT2i, plans predominantly covered empagliflozin (4744 plans [99.8%]) and dapagliflozin (4220 plans [88.8%]). Almost all SGLT2i were restricted (97.1%-99.8%), primarily due to tier level 3 or higher (97.1%-99.8%). Finerenone was universally covered (4754 plans [100%]) but nearly all plans enforced tier level 3 or higher (99.3%), and most required prior authorization (61.8%) (Table 1).

**Table 1.** Cardiovascular-Kidney-Metabolic Therapy Coverage Restrictions Under Quarter 3 2023 Medicare Prescription Drug Plans

Medication^a^	Medicare prescription drug plans, No. (%) (N = 4754)^b^					
	Any coverage^c^	Unrestricted^d^^,^^e^	Any restriction^d^^,^^e^	Tier level ≥3^e^	Prior authorization^e^	Step therapy^e^
GLP1RA						
Dulaglutide	4675 (98.3)	87 (1.9)	4588 (98.1)	4550 (97.3)	966 (20.7)	362 (7.7)
Long-acting exenatide	4073 (85.7)	57 (1.4)	4016 (98.6)	4013 (98.5)	748 (18.4)	311 (7.6)
Short-acting exenatide	2694 (56.7)	57 (2.1)	2637 (97.9)	2635 (97.8)	725 (26.9)	173 (6.4)
Liraglutide	4558 (95.9)	84 (1.8)	4474 (98.2)	4437 (97.3)	939 (20.6)	402 (8.8)
Injectable semaglutide	4533 (95.4)	84 (1.9)	4449 (98.1)	4412 (97.3)	904 (19.9)	402 (8.9)
Oral semaglutide	4399 (92.5)	59 (1.3)	4340 (98.7)	4312 (98.0)	892 (20.3)	343 (7.8)
GIP/GLP1RA						
Tirzepatide	2392 (50.3)	5 (0.2)	2387 (99.8)	2360 (98.7)	342 (14.3)	243 (10.2)
SGLT2i						
Canagliflozin	1865 (39.2)	4 (0.2)	1861 (99.8)	1861 (99.8)	0	84 (4.5)
Dapagliflozin	4220 (88.8)	124 (2.9)	4096 (97.1)	4096 (97.1)	7 (0.2)	0
Empagliflozin	4744 (99.8)	125 (2.6)	4619 (97.4)	4619 (97.4)	0	7 (0.1)
Ertugliflozin	145 (3.1)	1 (0.7)	144 (99.3)	144 (99.3)	7 (4.8)	27 (18.6)
nsMRA						
Finerenone	4754 (100)	17 (0.4)	4737 (99.6)	4722 (99.3)	2940 (61.8)	5 (0.1)

Abbreviations: GIP, glucose-dependent insulinotropic polypeptide; GLP1RA, glucagon-like peptide-1 receptor agonist; nsMRA, nonsteroidal mineralocorticoid receptor antagonist; SGLT2i, sodium-glucose-cotransporter-2 inhibitor.

^a^ Medications not covered by Medicare are not presented: bexagliflozin, sotagliflozin, Wegovy (semaglutide), Saxenda (liraglutide), and Zepbound (tirzepatide). For each medication, the lowest available maintenance dose, absent initial titration doses, was chosen.

^b^ Coverage calculations are based on 2023 quarter 3 Part D plan formularies and drug benefit data nationwide unweighted by plan enrollment and not based on patient claims.

^c^ Percentages are calculated from total number of Medicare prescription drug plans (4754 plans).

^d^ Individual plans were considered restricted if 1 or more of prior authorization, step therapy, or cost sharing at tier level 3 or higher were required.

^e^ Percentages are calculated from the Any Coverage column.

GLP1RA and GIP and GLP1RA had median annual OOP costs more than $2000, although there were differences between medications (median [IQR] range, $2012 [$1825-$2034] to $2508 [$2288-$2538]). Median (IQR) annual OOP costs for SGLT2i ranged from $1501 ($1365-$1535) to $1619 ($1593-$1964), except for ertugliflozin ($983 [$951-$1516]). Median (IQR) annual OOP cost for finerenone was $1692 ($1629-$1980) (Table 2).

**Table 2.** Estimated Annual List Prices and Out-of-Pocket (OOP) Costs for Cardiovascular-Kidney-Metabolic Therapy Under Quarter 3 2023 Medicare Prescription Drug Plans

Medication^a^	Median (IQR), US $	
	Annual list price	Annual OOP cost^b^
GLP1RA		
Dulaglutide	13 055 (12 363-13 138)	2369 (2311-2387)
Long-acting exenatide	11 296 (10 807-11 361)	2204 (2047-2258)
Short-acting exenatide	10 843 (10 047-10 944)	2508 (2288-2538)
Liraglutide	9740 (9093-9811)	2012 (1825-2034)
Injectable semaglutide	12 828 (12 750-13 140)	2369 (2342-2388)
Oral semaglutide	12 249 (11 679-12 329)	2373 (2321-2389)
GIP/GLP1RA		
Tirzepatide	13 953 (13 648-14 364)	2415 (2395-2429)
SGLT2i		
Canagliflozin	7852 (7676-7947)	1619 (1593-1964)
Dapagliflozin	7412 (7079-7456)	1501 (1365-1535)
Empagliflozin	7758 (7267-7816)	1570 (1429-1594)
Ertugliflozin	3996 (3971-4068)	983 (951-1516)
nsMRA		
Finerenone	8110 (7983-8256)	1692 (1629-1980)

Abbreviations: GIP, glucose-dependent insulinotropic polypeptide; GLP1RA, glucagon-like peptide-1 receptor agonist; nsMRA, nonsteroidal mineralocorticoid receptor antagonist; SGLT2i, sodium-glucose-cotransporter-2 inhibitor.

^a^ Medications not covered by Medicare are not presented: bexagliflozin, sotagliflozin, Wegovy (semaglutide), Saxenda (liraglutide), and Zepbound (tirzepatide). For each medication, the lowest available maintenance dose, absent initial titration doses, was chosen.

^b^ Projected annual OOP cost under 2023 Part D coverage benefits: deductible of $505, initial coverage phase until total drug costs reach $4660, coverage gap (beneficiaries pay 25% of total drug costs for brand-name drugs) until $11 206 in total drug costs are met, followed by catastrophic coverage (5% beneficiary copay) for remainder of the year. Insurance plan premiums and patient assistance plans such as Low Income Subsidy were not included in projections. Costs were calculated across plans covering the medication.

## Discussion

Despite availability of various CKM therapies, uptake in eligible populations remains poor.^2^ Our study highlights possible causes, notably (1) incomplete coverage, (2) burdensome restrictions, and (3) high annual OOP costs exceeding $1500 and $2000 for nsMRA or SGLT2i and GLP1RA or GIP/GLP1RA, respectively.

To improve access and equity, the recently passed Inflation Reduction Act (IRA) will cap Medicare OOP drug costs at $2000 per year.^4^ Although Medicare will negotiate prices for selected medications under the IRA, drugs fewer than 9 years from initial approval are excluded, limiting cost containment for most CKM medications.^4^ Additionally, Medicare may face future financial challenges in addressing CKM health due to expanding indications for these medications, such as GLP1RA for treatment of atherosclerotic cardiovascular disease in the absence of diabetes.^5^ At present, Medicare is legally prohibited from covering medications specifically indicated for obesity such as Wegovy (semaglutide) and Zepbound (tirzepatide). If semaglutide were used in all eligible Medicare beneficiaries for weight loss, spending on this treatment alone would exceed the entire Part D budget.^6^ How these factors will impact patient access to CKM therapies in the future remains uncertain.

Study limitations include projecting estimates of OOP costs based on individual plans and not patient claims, which may not reflect differences in enrollment or utilization among different plans. Estimates exclude insurance plan premiums and could not account for patient assistance programs. Additionally, we calculated OOP cost for each individual medication, and therefore actual OOP costs may be higher for patients taking multiple medications.

## Notes

Supplement 1.
eMethods.

Supplement 2.
Data Sharing Statement
