# Supplementary material for: Medicare Coverage and Patient Out-of-Pocket Costs for Cardiovascular-Kidney-Metabolic Medications
Source: JAMA Netw Open. 2024 May 21;7(5):e2412437. doi: 10.1001/jamanetworkopen.2024.12437 (PMC11109768; doi:10.1001/jamanetworkopen.2024.12437)
Supplement: Supplement 1. — eMethods. [file jamanetwopen-e2412437-s001.pdf]

Supplemental Online Content

Young GM, Bansal K, Riello III, RJ .Medicare coverage and patient out-of-pocket costs for cardiovascular-kidney-metabolic medications. *JAMA Netw Open*. 2024;7(5):e2412437. doi:10.1001/jamanetworkopen.2024.12437

eMethods.

This supplemental material has been provided by the authors to give readers additional information about their work.

© 2024 Young GM et al. *JAMA Network Open.*

eMethods

Newly approved medications not yet covered by Medicare were omitted. Injectable semaglutide (Ozempic), liraglutide (Victoza) and tirzepatide (Mounjaro) formulations for treatment of diabetes were included in this analysis.

Plan coverage and restrictions were analyzed across all Medicare Prescription Drug Plans. Individual plans were considered restricted if prior authorization, step therapy, or cost sharing at tier level ≥3 were required. Annual out-of-pocket costs were estimated for each plan covering a particular therapy. Monthly out-of-pocket costs during the initial coverage phase for each plan were used to project annual out-of-pocket costs under the 2023 Medicare Part D benefit ($505 deductible, initial coverage phase until total drug costs reach $4,660, coverage gap where beneficiaries pay 25% of total drug costs, followed by 5% copay during catastrophic coverage).

© 2024 Young GM et al. *JAMA Network Open.*
