# Supplementary material for: Medicare Coverage and Patient Out-of-Pocket Costs for Cardiovascular-Kidney-Metabolic Medications
Source: JAMA Netw Open. 2024 May 21;7(5):e2412437. doi: 10.1001/jamanetworkopen.2024.12437 (PMC11109768; doi:10.1001/jamanetworkopen.2024.12437)
Supplement: Supplement 2. — Data Sharing Statement [file jamanetwopen-e2412437-s002.pdf]

Data Sharing Statement

Young. Medicare Coverage and Patient Out-Of-Pocket Costs for Cardiovascular-Kidney-Metabolic Medications. *JAMA Netw Open*. Published May 21, 2024. doi:10.1001/jamanetworkopen.2024.12437

Data
Data available: Yes
Data types: Data (not involving human participants)
How to access data: https://www.cms.gov/research-statistics-data-and-systems/files-for-order/nonidentifiabledatafiles/prescriptiondrugplanformularypharmacynetworkandpricinginformationfiles
When available: With publication

Supporting Documents
Document types: None

Additional Information
Who can access the data: anyone requesting the data
Types of analyses: for any purpose
Mechanisms of data availability: without investigator support
